# The interrelation of parental alcohol use, parental practices, and binge drinking among secondary and high school students: analysis of a national survey

**DOI:** 10.3389/fpubh.2025.1662188

**Published:** 2025-10-28

**Authors:** Raquel Mondragón Gómez, Jorge A. Villatoro Velázquez, Maria Elena Medina-Mora Icaza, Emilia Lucio y Gómez Maqueo

**Affiliations:** ^1^School of Psychology, National Autonomous University of Mexico (UNAM), Mexico City, Mexico; ^2^Ramón de la Fuente Muñiz National Institute of Psychiatry (INPRFM), Mexico City, Mexico; ^3^Centro de Investigación en Salud Mental Global INPRFM UNAM, Mexico City, Mexico; ^4^Global Studies Seminar, School of Medicine, National Autonomous University of Mexico (UNAM), Mexico City, Mexico

**Keywords:** intergenerational transmission of alcohol use, parental problematic drinking, adolescent binge drinking, parenting practices, national probabilistic survey

## Abstract

**Introduction:**

Understanding the role of parenting practices and their potential interaction effect between parental and adolescent alcohol use is critically important for informing and developing interventions aimed at preventing binge drinking among adolescents.

**Objective:**

To evaluate the effect of parental problematic alcohol consumption and parenting practices on alcohol use among students, using a nationally representative sample of adolescents in Mexico.

**Methodology:**

A secondary analysis was conducted using the National Survey on Drug Use among Students, based on data from 114,364 middle and high school students. A multivariable analysis of prevalence ratios (PR) was conducted using generalized linear models (GLM) with log-link and binomial distribution. From this global analysis, the nlcom command was used to compare parenting practices within each category of parental problematic alcohol use.

**Results:**

The results indicated a higher risk of binge drinking among students who reported a father with problematic alcohol use and perceived negative supervision or lack of parental involvement (PR = 1.63 [95% CI: 1.43–1.86]; PR = 1.36 [1.19–1.56], respectively); among those who reported problematic use in both parents and perceived negative supervision (PR = 1.32 [95% CI: 1.06–1.64]); similarly, among those whose parents did not report problematic use, but perceived negative supervision or negligent parenting practices (PR = 1.50 [95% CI: 1.41–1.59]; PR = 1.13 [95% CI: 1.04–1.24], respectively), and when they perceived no parental involvement or encouragement (PR = 1.44 [95% CI: 1.35–1.53]; PR = 1.12 [95% CI: 1.04–1.24], respectively).

**Discussion:**

The findings highlight the importance of developing and strengthening prevention efforts that promote positive parenting practices and enhance parents’ understanding of how their alcohol use affects their children’s behavior.

## Introduction

### Parenting practices and their influence on adolescent alcohol use

Parenting practices encompass behaviors that mothers and fathers employ to influence the development of their children, such as discipline, supervision, positive involvement, or emotional support (1, 2). In recent decades, numerous studies have shown that family environment and parenting practices play a key role in preventing alcohol use in adolescents.

For example, family communication has been significantly associated with lower alcohol use among adolescents (3). In this study, which reported a 26.1% prevalence of alcohol use among adolescents, the quality of intrafamilial communication functioned as a significant protective factor. Complementarily, Ortega and Jódar (4) found that affection, communication, and behavioral control exert a preventive effect, while practices such as criticism, rejection, or lax discipline increase the risk of use.

This pattern was also documented by Musitu Ochoa et al. (5), who emphasized that family communication, household functioning, and adolescent self-esteem create a protective network against alcohol use. Their findings suggest that when effective communication and a healthy family environment mediated by positive self-esteem are present, the likelihood of adolescents initiating or maintaining alcohol consumption decreases.

Several studies showed that parental norms and supervision were associated with lower lifetime alcohol use, as well as with reduced likelihood of recent consumption and intoxication (6). Likewise, adolescents who perceived a high level of parental involvement, together with clear limits regarding substance use, were less likely to engage in risky behaviors (7). Along the same lines, active supervision—including knowing children’s friends, setting clear expectations, and monitoring daily activities—was linked to lower rates of alcohol use (8).

Through a meta-analysis, Pinquart and Gochaliyev (9) identified that parental warmth was not only associated with lower alcohol use, but also with reduced tobacco and cannabis use in early adolescence. Complementarily, Yockey et al. (10) reported that the lack of academic supervision and expressions of pride toward children increased the risk that adolescents would drive under the influence of alcohol or other drugs.

In this regard, in addition to parenting practices, the evidence on parenting styles has shown a clear influence on alcohol use. For example, maternal permissive parenting has been found to increase the risk of binge drinking episodes during adolescence, whereas an authoritative parenting style reduces it (11, 12). Taken together, these findings reinforced the idea that parenting practices and styles not only shaped the emotional climate of the home but also functioned as external regulatory mechanisms that influenced adolescents’ decisions regarding substance use.

### Parental alcohol use and its consequences for adolescents

Several studies have documented that problematic alcohol use by parents has significant effects on the mental health and behavior of their children, including a higher prevalence of anxiety, depressive symptoms, attention problems such as ADHD, disruptive behaviors, increased risk of accidents (13–15), and binge drinking from early ages (16–19).

One of the most consistent explanations for this relationship is known as the Intergenerational Transmission of Alcohol Use (ITAU), a concept that refers to the continuity of alcohol-related patterns and traits across generations (20, 21). This phenomenon has been addressed from both genetic and psychosocial perspectives, with the latter being especially useful for understanding environmental mechanisms that mediate this transmission.

From this perspective, Orford and Velleman (22) proposed two types of environmental mechanisms: specific (SEM) and general (GEM). SEM is based on Social Learning Theory (23), which posits that adolescents may adopt drinking behaviors by observing their parents’ behavior, particularly when these behaviors are positively reinforced. In this regard, Akers et al. (24) argued that children tend to imitate behaviors they perceive as rewarded; thus, if they see that their parents’ alcohol use yields social or emotional benefits, they are more likely to adopt the behavior.

GEM, on the other hand, focuses on the deterioration of the general family environment due to parental alcohol use. According to Velleman (25), alcohol use may interfere with multiple dimensions of the family setting, affecting the quality of parenting practices. The most affected aspects include supervision, discipline, and emotional stability at home, all key elements in preventing adolescent alcohol use.

In summary, the evidence suggests that parental alcohol use not only serves as a direct behavioral model for children but also disrupts the structural and emotional conditions of the home, thereby increasing the likelihood that adolescents will adopt similar consumption patterns.

### The interplay between parental alcohol use, parenting practices, and adolescent alcohol use

For several decades, it has been recognized that problematic alcohol use in mothers and fathers not only directly influences their children but also affects the quality of parenting practices and the overall structure of family life. In the 1980s, Wolin (26) noted that presence of an alcohol use disorder in one or both parents increased the likelihood that their children would also develop alcohol-related problems, especially when family rituals—such as shared meals, celebrations, or weekends—were disrupted by episodes of heavy drinking.

This family disruption not only affects daily life but also alters the ways in which parents relate to their children. Kandel (27), for instance, observed that as substance use increases, parental supervision decreases, punitive disciplinary practices become more common, and parental conflicts related to child-rearing increase. Similarly, Chassin et al. (28) found that parents with problematic alcohol use tend to monitor their children’s activities less, which increases the likelihood that adolescents associate with peers who also use substances.

More recent studies have reinforced these findings. Jackson et al. (29) and Keer et al. (30) reported that more frequent drinking and episodes of intoxication by both parents are associated with lower parental supervision, reduced family cohesion, greater tolerance for early alcohol use by children, and a general decline in the quality of the family environment. This combination of factors may foster the presence of violence, neglect, and the absence of a safe and protective environment.

Taken together, this body of research shows that parental alcohol use and parenting practices are not isolated elements but interrelated dimensions that, when they coincide, can amplify the risk of adolescent alcohol use. Despite this evidence, most studies tend to analyze these factors independently, without sufficiently exploring how they interact. Understanding this interaction is essential for deepening our grasp of the mechanisms of Intergenerational Transmission of Alcohol Use (ITAU) and for designing more comprehensive and effective interventions.

Based on these considerations, the present study analyzes the relationship between parenting practices and binge drinking among adolescent students, comparing those with and without a parental history of alcohol use, based on data from a national representative survey.

## Methods

### Study design

Descriptive, comparative, and correlational study through a secondary analysis of a national probabilistic survey conducted among the school population [see (31) for more details on the study].

### Participants and sample

The National Survey of Drug Use in Students (ENCODE) was conducted by the Ramón de la Fuente Muñiz National Institute of Psychiatry (INPRFM), the National Center for Addiction Prevention and Control (CENADIC), and the Ministry of Public Education (SEP). The study was carried out in the second semester of 2014 with a random and representative sample of secondary and high school students, using a systematic, stratified, and cluster sampling method across the 32 states of Mexico. The sample size was estimated considering a 20% non-response rate, with a confidence level of 95%. The final sample included 114,364 students (31).

### Variables

*Parental Alcohol Consumption History (HCPA):* Refers to students’ perceptions of their parents’ alcohol consumption. It was assessed through two questions: 1. “Does your father have problems due to his alcohol consumption?” and 2. “Does your mother have problems due to her alcohol consumption?” For the purposes of this study, these questions were grouped into the HCPA variable with four categories:

- HCPA-N = Neither parent has problematic alcohol consumption
- HCPA-P = Only the father has problematic alcohol consumption
- HCPA-M = Only the mother has problematic alcohol consumption
- HCPA-A = Both parents have problematic alcohol consumption.

Binge drinking: This variable was measured with an item asking whether students had consumed five or more alcoholic drinks on a single occasion within the 30 days preceding the survey.

*Parenting Practices:* To evaluate parenting practices, the Alabama Scale (32–34) adapted in Mexico was used. Each item consisted of 4 frequency response options. The responses from each area were summed, and the total was divided by the number of items to obtain an average score. The average score was recoded, with values ranging from 1.00 to 1.50 classified as 0 (skill not present) and values from 1.51 to 4.00 classified as 1 (skill present).

The areas assessed by the scale are as follows:

- *Positive Parental Involvement:* Consists of 9 items and measures whether caregivers spend time with their children and show interest and affection in positive interactions (Standardized Cronbach’s alpha = 0.89).
- *Supervision:* Consists of 3 items and measures whether caregivers are aware of the children’s friends, places they frequent, and their activities and behaviors, both inside and outside the home (Standardized Cronbach’s alpha = 0.74).
- *Parental Encouragement:* Consists of 5 items and measures the use of positive contingencies, skills development, and reinforcement (Standardized Cronbach’s alpha = 0.86).
- *Neglect:* Consists of 2 items, measuring rule-setting and whether inappropriate behaviors are discouraged through negative (non-physical) sanctions appropriate to the child’s behavior and age (Standardized Cronbach’s alpha = 0.71).

*Sociodemographic* Var*iables:* Gender (male, female), presence of mother in the household (yes, no), presence of father in the household (yes, no), level of education (secondary or high school), type of community (urban or rural), perceived socioeconomic status(lower, lower middle, middle, middle high and high, school status (studied last year, did not studied last year), employment status work last year, did not worked last year), and membership in an indigenous-speaking community (yes, no). These variables were used as covariates in the data analysis. A more comprehensive definition of the covariates is presented in the Supplementary material as referenced in Table 2.

### Data analysis

All analyses were performed using STATA v.16 software. The survey’s sampling design was taken into account using the *svy* command in Stata. The clustering variable was the school group, stratification was defined by state and educational level, and the probability of selection was incorporated through each individual’s sampling weight.

Based on these elements, a multivariable analysis of prevalence ratios (PR) was performed using generalized linear models (GLM) with a log-link and binomial distribution. From this global analysis, the nlcom command was used to obtain, within each category of parent’s problematic consumption, the comparison of each parenting skill, with the following interactions as predictors: (1) HCPA and negative supervision, (2) HCPA and positive parental involvement, (3) HCPA and parental encouragement, and (4) HCPA and negligence. In addition, the following variables were included as covariates or control variables: presence of the mother in the household, presence of the father in the household, gender, education level, type of community, perceived socioeconomic status, school status, employment status, and indigenous status. The dependent variable was whether or not the student had binge drinking.

### Ethical considerations

The project was approved by the Ethics Committee for Research at the “Ramón de la Fuente Muñiz” National Institute of Psychiatry with approval number CEI/C/020/2014. Students could choose whether or not to complete the questionnaire, and they were informed that they could stop responding at any time if they wished.

## Results

### Participant characteristics

Women who participated in the study (50.2%), 61.4% were secondary school students, 81.2% were full-time students, and 14.3% were also engaged in work activities. It was also found that 21% reported a low perceived socioeconomic status (SES), and 19.1% reported a high SES. The majority were from urban communities (85.2%), and only 11.1% belonged to an indigenous community. Regarding the men, 62.9% were attending secondary school, 78.2% were full-time students, and 29.4% were also engaged in work activities. The majority had a middle-high SES (22.6%), 84.5% were from urban communities, and 11.7% belonged to an indigenous community (Table 1).

**Table 1:** Sociodemographic characteristics.

	Female 58,083 50.2%		Male 56,281 49.8%		Total	
	n	%	n	%	n	%
Educational Level						
Secondary School	28,825	61.4	28,577	62.9	57,402	62.2
High School	29,258	38.6	27,704	37.1	56,962	37.8
Socioeconomic Index						
Low	11,289	21.0	8,843	16.8	20,132	18.9
Lower-middle	11,016	20.5	10,180	19.3	21,196	19.9
Middle	10,234	19.0	10,450	20.0	20,684	19.5
Upper-middle	10,784	20.4	11,662	22.6	22,446	21.5
High	10,399	19.1	11,013	21.2	21,412	20.1
Community						
Rural	6,068	14.8	6,299	15.5	12,367	15.1
Urban	52,015	85.2	49,982	84.5	101,997	84.9
Previous Year (Study)						
Studied Full Time	46,566	81.2	43,318	78.2	89,884	79.7
Did Not Study	10,448	18.8	11,864	21.8	22,312	20.3
Previous Year (Work)						
Did Not Work	49,258	85.7	39,397	70.6	88,655	78.2
Worked	7,932	14.3	15,849	29.4	23,781	21.8
Population Type						
Indigenous	6,165	11.1	6,378	11.7	12,543	11.4
Non-Indigenous	51,918	88.9	49,903	88.3	101,821	88.6

### Binge drinking in students with a father with problematic drinking

In this group, it was found that students reporting negative supervision had a higher risk of binge drinking compared to those who did not perceive negative supervision (PR = 1.63 [95% CI 1.43–1.86]). Additionally, students who felt that their fathers were not involved with them had a 36% higher risk of binge drinking (PR = 1.36 [95% CI 1.19–1.56]). As for parental encouragement and neglect, these factors did not impact the students’ binge drinking (Table 2).

**Table 2:** Multivariable prevalence ratios between parental alcohol consumption history and parental practices with binge drinking in a national sample of 7th to 12th grade students*.

	No parental problematic drinking					Father problematic drinking					Mother problematic drinking					Both parents problematic drinking				
	n	%	PR	*p*	CI 95%	n	%	PR	*p*	CI 95%	n	%	PR	*p*	CI 95%	n	%	PR	*p*	CI 95%
Negative supervision																				
Absent	9,509	12.7	1			1709	18.3	1			396	35.6	1			489	32	1		
Present	3,917	19.7	1.50	***p* < 0.001**	1.41–1.59	715	27.3	1.63	***p* < 0.001**	1.43–1.86	186	42.7	1.15	0.321	0.87–1.50	236	43.8	1.32	**0.014**	1.06–1.64
Positive involvement																				
Present	7,406	12.2	1			1,117	17	1			274	34.2	1			384	34.6	1		
Absent	5,671	17.7	1.44	***p* < 0.001**	1.35–1.53	1,212	23.6	1.36	***p* < 0.001**	1.19–1.56	303	42	1.28	0.089	0.96–1.71	320	35.6	1.14	0.272	0.90–1.44
Parental encouragement																				
Present	7,344	12.8	1			1,190	18.9	1			285	35.9	1			395	35.8	1		
Absent	5,907	16.2	1.12	***p* < 0.001**	1.06–1.20	1,199	21.7	1.05	0.485	0.92–1.20	309	41.2	0.96	0.777	0.71–1.29	310	33.7	0.96	0.765	0.90–1.44
Negligence																				
Absent	11,442	13.7	1			2004	19.3	1			475	30	1			598	35.2	1		
Present	2098	17.1	1.13	**0.003**	1.04–1.24	429	18	0.99	0.95	0.84–1.18	124	32	1.08	0.617	0.79–1.49	130	33.4	1.05	0.737	0.80–1.37

*Adjusted results for the variables: Sex, presence of the mother in the household, presence of the father in the household, educational level (secondary or high school), type of community (urban or rural), perceived socioeconomic status, school status, employment status, and membership in an indigenous language community. The complete adjusted analysis can be consulted in the Supplementary materials, https://drive.google.com/file/d/1ScQbziKS40bEiL4J3ZgrWFei0jK908F1/view?usp=drive_link. The bold probabilities (p) correspond to the predictors with statistical significance.

### Binge drinking in students with a mother with problematic drinking

In the students of this group (perception of maternal consumption) no statistically significant differences were found in any parenting practice.

### Binge drinking in students with problematic drinking in both parents

In this group, it was found that students who reported negative parental supervision had a higher risk of binge drinking compared to those who did not report such supervision (PR = 1.32 [95% CI 1.06–1.64]). For the other parenting practices, binge drinking was similar among the students.

### Binge drinking in students with non-problematic drinking parents

In this section, it was found that those who reported the presence of negative supervision had a 50% higher risk of binge drinking compared to those who did not report it (PR = 1.50 [95% CI 1.41–1.59]). Similarly, when parental neglect was perceived, the risk of binge drinking was higher compared to when it was not perceived (PR = 1.13 [95% CI 1.04–1.24]). Additionally, when students did not perceive parental involvement and encouragement, the risk of binge drinking was higher (PR = 1.44 [95% CI 1.35–1.53]; PR = 1.12 [95% CI 1.04–1.24], respectively; Table 2).

## Discussion

The results indicated variations in the risk of binge drinking among students in relation to parental alcohol consumption and parenting practices. Regarding binge drinking in the group without parental alcohol consumption reports, differences were found in all parenting areas. This finding aligns with research indicating that parenting practices are a protective factor against alcohol and other substance use, especially when there is no problematic alcohol use among parents (35–39).

On the other hand, for students whose fathers had problematic alcohol use, the main variations in binge drinking were observed regarding supervision and involvement. Another finding was that when both parents had problematic drinking, the risk of binge drinking was higher in the presence of negative supervision. These results align with Latendresse et al. (40), who identified that parental supervision and discipline are potential mediators in the association between parental and adolescent alcohol use. Additionally, Mahedy et al. (41) indicated that parental supervision mediated the relationship between parents’ and children’s alcohol consumption. In other words, parental alcohol use implied less supervision, which facilitated adolescents’ interactions with peers who used substances, particularly with higher consumption levels among teenagers.

The results in the groups with problematic alcohol consumption in the father and both parents are consistent with findings suggesting that parental problematic alcohol use interferes with the development and implementation of certain parenting skills through various pathways. For example, the immediate effects of alcohol use on the Central Nervous System could affect positive parenting practices due to inhibition of prefrontal activity, impaired judgment, decision-making abilities, and motor coordination, among others (42–44). Similarly, parental care has been documented to rely on the interaction of multiple brain circuits that integrate functions such as motivation, empathy, reward, stress regulation, and cognitive control, all of which may be altered by parental stress, depression, or substance use (45).

It has also been noted that problematic alcohol use and drug use can impact parenting abilities in various ways. One of these ways is that alcohol use could act as a barrier to the acquisition and development of parenting practices. Additionally, drug use might influence the perception of parents regarding their children’s needs. Another aspect involves deficits in stress-coping skills related to motherhood, and another variable is attachment problems between the child and mother (46). Moreover, parenting practices could be affected by other processes directly associated with indicators of problematic or dependent alcohol use. Mayes and Truman (47) noted that mothers with substance abuse issues might prioritize satisfying their addiction over the well-being of themselves and their children. Neger and Prinz (43) suggested that substance abuse in adults could also relate to a decreased enjoyment of parenting, further affecting it through a continuous feedback loop between substance use and deficits in emotional regulation.

In contrast, for students with maternal problematic drinking, no statistically significant differences in binge drinking were observed. This finding contradicts other studies suggesting that supervision (41, 42), discipline (48), or shared activities (40) may be mediating variables between mothers’ and children’s alcohol use, and even in other behaviors such as gambling (49). Based on these results, it can be hypothesized that the parenting practices evaluated in this study were not as affected by maternal alcohol consumption due to the strong cultural and social importance of parenting practices for Mexican mothers. Furthermore, other parenting variables, such as bonding skills (50), may hold greater relevance for Mexican mothers.

### Limitations

It is important to mention that this study has some limitations. The main one is that this is a cross-sectional study, meaning the data should be interpreted in terms of association and not causality. Additionally, this characteristic does not allow us to identify how long the parents have been dealing with problematic alcohol consumption, which could be key to understanding the relationship between parental and child alcohol consumption. It is certainly possible and worthwhile to further explore the information presented here using alternative data analysis approaches and by incorporating additional variables of interest, such as parental mental health characteristics (e.g., anxiety or depression), which also influence the execution of parenting practices (51).

For future research, it is important to explore more indicators of parental alcohol use (dependence, daily use, fights between parents during consumption, etc.), as well as include aspects such as rule-setting regarding alcohol consumption, specific communication about alcohol use, permissiveness toward alcohol use, or the provision of alcohol (41, 46).

Despite these limitations, it is important to highlight that this study is one of the first to address the implications of parenting in the context of adolescent alcohol consumption with a probabilistic sample and national representation.

## Conclusion

The results indicated variations in the risk of binge drinking among students based on parenting and parental alcohol use history, except for the group with maternal problematic consumption, where parenting practices did not appear to affect adolescent binge drinking.

The findings of this research suggest that in scenarios where parental problematic alcohol consumption exists, and there is adequate implementation of parenting practices, such as supervision and parental involvement, the risk of binge drinking in adolescents may be reduced. Additionally, they reinforce the importance of appropriately implementing parenting practices to prevent binge drinking in adolescents.

These findings suggest that health agencies, schools, or addiction treatment centers should take preventive actions, such as providing psychoeducational materials or specific courses for parents, to offer information on the role they can play in their children’s alcohol use.

## Data Availability

The data analyzed in this study is subject to the following licenses/restrictions: this is a investigation research project. Requests to access these datasets should be directed to Jorge Villatoro, ameth@inprf.gob.mx.
