# Supplementary material for: The interrelation of parental alcohol use, parental practices, and binge drinking among secondary and high school students: analysis of a national survey
Source: Front Public Health. 2025 Oct 28;13:1662188. doi: 10.3389/fpubh.2025.1662188 (PMC12602431; doi:10.3389/fpubh.2025.1662188)
Supplement: Supplementary file 1 [file Supplementary_file_1.pdf]

Supplementary material
Model of excessive alcohol consumption in students

Variables		Binge Drinking				
		n	%	PR	*p*	CI 95%
**Parental Alcohol Consumption History**	***None***	13901	14.2%	1		
	Father	2491	20.1%	1.5	**p < 0.001**	1.30-1.72
	Mother	622	37.9%	3.64	**p < 0.001**	2.68-4.95
	Both	749	34.7%	4.16	**p < 0.001**	3.22-5.38
**Negative Supervision**	***No***	12103	13.9%	1		
	Yes	5054	21.5%	1.66	**p < 0.001**	1.53-1.80
**Positive Parental Involvement**	***Yes***	9181	13.3%	1		
	No	7506	19.3%	1.56	**p < 0.001**	1.44-1.69
**Parental Encouragement**	***Yes***	9214	14.1%	1		
	No	7725	17.7%	1.15	**p < 0.001**	1.07-1.24
**Neglect**	***No***	14519	15.0%	1		
	Yes	2781	18.9%	1.17	**0.004**	1.05-1.30
**Sex**	***Female***	8295	14.3%	1		
	Male	9468	16.8%	1.05	**0.083**	0.99-1.12
**Educational level**	***Secondary***	4559	7.9%	1		
	High school	13204	23.2%	3.29	**p < 0.001**	3.00-3.60
**Perceived socioeconomic status**	***Lower***	3074	15.3%	1		
	Lower Middle	3273	15.4%	1.1	**0.022**	1.01-1.20
	Middle	3188	15.4%	1.23	**p < 0.001**	1.12-1.34
	Middle high	3396	15.1%	1.27	**p < 0.001**	1.16-1.39
	High	3744	17.5%	1.51	**p < 0.001**	1.37-1.65
**Type of community**	***Rural***	1366	11.0%	1		
	Urban	16397	16.1%	1.19	**0.030**	1.01-1.39
**School status**	***Studied last year***	13466	15.0%	1		

	Did not studied last year	3991	17.9%	1.12	**0.002**	1.04-1.20
**Employment status**	***Did not worked last year***	12153	13.7%	1		
	Work last year	5346	22.5%	1.57	**p < 0.001**	1.47-1.67
**Membership in an indigenous-speaking family/group**	***Yes***	1571	12.5%	1		
	No	16192	15.9%	1.15	**0.006**	1.04-1.028
**Do you live with your mother at home?**	***No***	1553	20.6%	1		
	Yes	16210	15.2%	0.88	**0.017**	0.79-0.97
**Do you live with your father at home?**	***No***	5199	18.2%	1		
	Yes	12564	14.6%	0.82	**p < 0.001**	0.77-0.87

Definition of Covariates:

Variable	Definition / Measurement	Categories	Source
**Sex**	**Self-reported sex**	**Female; Male**	
**Educational Level**	**School grade attended**	**Secondary = 7th–9th grade; High school = 10th–12th grade**	
**Perceived Socioeconomic Status (PSS)**	**Oetting & Beauvais scale with 11 items; quintile distribution**	**Low; Lower-middle; Middle; Upper-middle; High**	**Oetting & Beauvais[1]; applied in several studies in Mexico[2,3]**
**Type of Community**	**Population size of locality**	Rural = ≤2,499 inhabitants; Urban = ≥2,500 inhabitants	**National Institute of Statistics and Geography (INEGI)[4]**
**School Status**	**Item asking if the student studied most of the past year**	**Studied last year; Did not study last year**	
**Employment Status**	**Item asking if the student worked most of the past year**	**Worked last year; Did not work last year**	
**Membership in an Indigenous-Speaking Community**	**Living in a household where at least one member speaks an Indigenous language, or the student speaks it**	**Indigenous; Non-Indigenous**	**National Population Council (CONAPO 2020)[5]**
**Presence of Mother in the Household**	**Item: *Do you live with your mother at home?***	**Yes; No**	
**Presence of Father in the Household**	**Item: *Do you live with your father at home?***	**Yes; No**	

References of supplementary material:

1. Oetting ER, Beauvais F. Common elements in youth drug abuse: peer clusters and other psychosocial factors. *J Drug Issues*. 1987;17(2):133-51. doi:10.1177/002204268701700202.
2. Rojas E, Fleiz C, Medina-Mora ME, Morón-Domenech M. Consumo de alcohol y drogas en la población estudiantil de la ciudad de Pachuca Hidalgo. *Salud Publica Mex*. 1999;41(4):297-301.
3. Villatoro J, Oliva N, Mujica R, Fregoso D, Bustos M, Medina-Mora ME. Panorama actual del consumo de sustancias en estudiantes de la Ciudad de México. México, D.F.: Instituto Nacional de Psiquiatría Ramón de la Fuente Muñiz; Administración Federal de Servicios Educativos en el Distrito Federal; Instituto para la Atención y Prevención de las Adicciones; 2015. Available from: https://www.researchgate.net/publication/283644601_Panorama_Actual_del_Consumo_de_Sustancias_en_Estudiantes_de_la_Ciudad_de_Mexico [accessed 2025 Sep 01].
4. Instituto Nacional de Estadística y Geografía (INEGI). Diccionario de datos de la cartografía geoestadística histórica de México. México: INEGI; 2024. p. 33. Available from: https://www.inegi.org.mx/contenidos/productos/prod_serv/contenidos/espanol/bvinegi/productos/nueva_estruc/889463919988.pdf
5. Consejo Nacional de Población (CONAPO). Población indígena en México: características sociodemográficas 2020 [infografía]. México: CONAPO; 2020. Available from: https://www.gob.mx/conapo/documentos/poblacion-indigena-en-mexico-caracteristicas-sociodemograficas-2020
